# The Role of the CHA_2_DS_2_-VASc Score in predicting postoperative Atrial Fibrillation after pulmonary resection

**DOI:** 10.12669/pjms.42.1.12822

**Published:** 2026-01

**Authors:** Hasan Ekrem Camas, Serap Yildirim, Suleyman Emre Akin, Bayram Ali Uysal

**Affiliations:** 1 Hasan Ekrem Camas Assistant Professor, Faculty of Medicine, Department of Thoracic Surgery, Suleyman Demirel University, Isparta, Turkiye; 2 Serap Yildirim Faculty of Medicine, Department of Thoracic Surgery, Suleyman Demirel University, Isparta, Turkiye; 3 Suleyman Emre Akin Assistant Professor, Faculty of Medicine, Department of Thoracic Surgery, Suleyman Demirel University, Isparta, Turkiye; 4 Bayram Ali Uysal Associate Professor, Faculty of Medicine, Department of Cardiology, Suleyman Demirel University, Isparta, Turkiye

**Keywords:** Lung, Atrial Fibrillation, Risk Factors, Thoracic Surgery

## Abstract

**Objective:**

CHA_2_DS_2_-VASc is a validated scoring system used to calculate the risk of stroke and thrombus in atrial fibrillation (AF) conditions. In this study, we aimed to evaluate the relationship between the development of postoperative atrial fibrillation (PAF) and CHA_2_DS_2_-VASc score in patients undergoing elective anatomical lung resection.

**Methodology:**

In this retrospective cohort study, the records of patients who underwent elective anatomic lung resection at a single center between January 2015 to December 2024 were reviewed. The CHA_2_DS_2_-VASc score of all patients was calculated. Mann-Whitney U, Chi-square and ROC analyses, as well as binary logistic regression, were used for statistical analysis.

**Results:**

Among one hundred patients (80 males), the incidence of PAF was 11%. Patients with PAF had significantly higher CHA_2_DS_2_-VASc scores (p<0.001). ROC analysis showed an AUC of 0.797 (p<0.001), with a cut-off of 1.5 yielding 90.9% sensitivity and 34.8% specificity. Logistic regression identified the CHA_2_DS_2_-VASc score as a significant predictor of PAF (OR=4.33, p=0.039).

**Conclusion:**

The CHA_2_DS_2_-VASc score may be a valuable tool not only to assess thromboembolic risk but also to predict the development of PAF after thoracic surgery. With this scoring, high-risk patients can be identified in advance, appropriate prophylactic measures can be taken, and postoperative morbidity and mortality can be reduced.

## INTRODUCTION

Atrial fibrillation (AF) is one of the most common types of serious arrhythmias in the postoperative period.^1^,^2^ The incidence of postoperative atrial fibrillation (PAF) varies depending on the type of surgical procedure performed.^3^ Although the highest incidence of PAF is observed in cardiovascular surgeries, it is most frequently observed in thoracic surgeries in non-cardiac surgical procedures. It has been reported that approximately 10%-20% of patients undergoing thoracic surgery develop PAF.^4^,^5^

The management of PAF is crucial to reduce mortality, morbidity, and associated hospitalization and treatment costs. The first step in this approach involves accurate identification of risk factors. According to various studies in the literature, major risk factors associated with the development of PAF include advanced age, male gender, hypertension (HT), ischemic heart disease, peripheral arterial disease, congestive heart failure (CHF), hypoxia, perioperative electrolyte imbalances, and extent of pulmonary resection.^6^-^8^

Especially in patients undergoing pulmonary resection, pneumonectomy, right-sided surgeries, hilar dissections, and the presence of an infected bronchial stump are considered among the important factors that trigger the development of PAF.^6^,^9^ The development of PAF is not only limited to rhythm disturbance but also predisposes to serious complications, including heart failure, stroke, systemic embolism, atrial thrombosis, and hypotension, leading to increased postoperative mortality, morbidity, and hospitalization.^4^,^6^,^10^

The CHA_2_DS_2_-VASc score is widely used to identify patients at high and intermediate risk for thromboembolic events in the presence of atrial fibrillation. The parameters included in this score are age, gender, HT, diabetes mellitus (DM), cerebrovascular disease (CVD), vascular disease, and CHF.^11^,^12^ Currently, there is no validated model for predicting the development of PAF in critically ill patients. However, some studies have shown that the CHA_2_DS_2_-VASc score may have predictive value not only for thromboembolic risk but also for the development of AF.^13^ Nevertheless, there is no sufficiently validated risk model for predicting the development of PAF after thoracic surgery.^14^

This study aimed to evaluate the relationship between the CHA_2_DS_2_-VASc score and the development of PAF in patients who underwent pulmonary resection. Based on the data obtained, the goal is to identify high-risk patients and take appropriate prophylactic measures to reduce morbidity and mortality.

## METHODOLOGY

This retrospective cohort study, reviewed the medical records of adult patients (aged ≥18 years) who underwent elective anatomical lung resection (including lobectomy and pneumonectomy) at a single center between January 2015 to December 2024. Patients with a prior diagnosis of chronic or paroxysmal AF, or those with documented transient AF episodes before surgery, were excluded from the analysis.

### Ethical Approval:

It was obtained from the Clinical Research Ethics Committee of Suleyman Demirel University (Approval No: 26.01.22-3/39),

### Data Collection and Definitions:

The occurrence of PAF was assessed from the initiation of surgery through postoperative day-7. All patients underwent continuous intraoperative electrocardiographic (ECG) monitoring, continuous ECG monitoring in the intensive care unit (ICU), and at least one daily 12-lead ECG during their hospitalization.The CHA_2_DS_2_-VASc score was calculated for each patient upon admission according to the following criteria: one point each for CHF, HT, DM, vascular disease, age 65-74 years, and female gender; two points each for a history of CVD and age ≥75 years.

### Statistical Analysis:

Statistical analyses were conducted using IBM SPSS Statistics for Windows, Version 27.0 (Armonk, NY: IBM Corp). Categorical variables were expressed as frequency and percentage; numerical variables as mean ± standard deviation and median (min-max). Normality was tested with the Kolmogorov-Smirnov test. Group comparisons used the Mann-Whitney U test and chi-square test. ROC analysis evaluated the predictive power of the CHA_2_DS_2_-VASc score, while binary logistic regression identified potential prognostic factors. A p-value <0.05 was considered statistically significant.

## RESULTS

AF was detected in 11 patients (11%) during the postoperative hospitalization period. Age and sex did not differ significantly between groups. However, HT (p=0.010), DM (p=0.003), vascular disease (p=0.036), and obesity (p=0.034) were more prevalent in patients with AF. No significant differences were found in CHF (p=0.363), CVD (p=0.725), smoking (p=0.525), or alcohol use (p=0.774). While certain lobectomies (right upper and left lower) were not performed in AF patients, operation type was not significantly associated with AF (p=0.120). Stage distribution approached significance (p=0.080), with higher proportions of stage three disease in the AF group. CHA_2_DS_2_-VASc scores were significantly higher in AF patients (p<0.001), with a median score of 2 (2.72±1.55) versus 1 (1.19±1.07) in the non-AF group. Length of hospital stay (p=0.409) and drainage volume (p=0.663) did not differ significantly (Table-I).

**Table-I:** General characteristics of patients according to postoperative atrial fibrillation (AF) status.

Feature	Category	Post-op AF None N (%)	Post-op AF Present N (%)	p-value
Gender	Female	19 (21,3)	1 (9,1)	0,340
	Male	70 (78,7)	10 (90,9)	
Heart Failure	No	86 (96,6)	10 (90,9)	0,363
	Yes	3 (3,4)	1 (9,1)	
Hypertension	No	66 (74,2)	4 (36,4)	0,010*
	Yes	23 (25,8)	7 (63,6)	
Diabetes	No	75 (84,3)	5 (45,5)	0,003*
	Yes	14 (15,7)	6 (54,5)	
Cerebrovascular accident	No	88 (98,9)	11 (100)	0,725
	Yes	1 (1,1)	0 (0)	
Vascular disease	No	78 (87,6)	7 (63,6)	0,036*
	Yes	11 (12,4)	4 (36,4)	
Cigarette smoking	No	33 (37,1)	3 (27,3)	0,525
	Yes	56 (62,9)	8 (72,7)	
Alcohol use	No	83 (93,3)	10 (90,9)	0,774
	Yes	6 (6,7)	1 (9,1)	
Obesity	No	86 (96,6)	9 (81,8)	0,034*
	Yes	3 (3,4)	2 (18,2)	
Method of Operation	Right lower lobectomy	17 (19,1)	0 (0)	0,120
	Right upper lobectomy	24 (27,0)	3 (27,3)	
	Left lower lobectomy	20 (22,5)	4 (36,4)	
	Left upper lobectomy	16 (18,0)	0 (0)	
	Pneumonectomy	12 (13,5)	4 (36,4)	
Tumor Stage	Benign	14 (15,7)	1 (9,1)	0,080
	Stage 1	29 (32,6)	2 (18,2)	
	Stage 2	30 (33,7)	3 (27,3)	
	Stage 3	16 (18,0)	5 (45,5)	
Mean ± SD (Median)	Age (years)	59,33 ± 11,98 (63)	64,63 ± 8,12 (65)	0,251
	CHA₂DS₂-VASc Score	1,19 ± 1,07 (1)	2,72 ± 1,55 (2)	0,001**
	Hospital Stay (days)	11,74 ± 7,18 (10)	12,90 ± 6,59 (11)	0,409
	Total Drainage (mm³)	990,44 ± 658,97 (850)	1345,45 ± 1182,46 (900)	0,663

* p < 0.05, significant according to chi-square analysis.

** p < 0.05, significant according to the Mann-Whitney U test.

**Fig.1:**
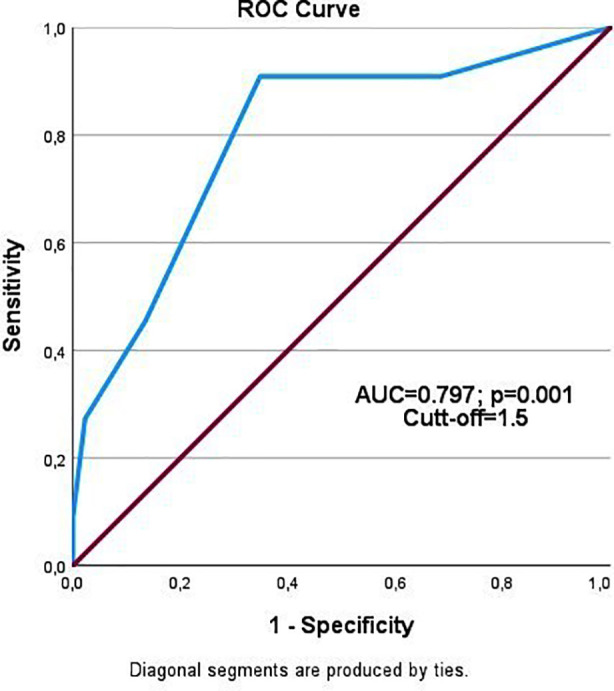
ROC curve for the CHA_2_DS_2_-VASc score. The area under the curve (AUC) was 0.797 (p<0.001), indicating a good predictive ability for postoperative atrial fibrillation.

Given the significant difference in CHA_2_DS_2_-VASc scores between patients with and without postoperative AF, ROC analysis was conducted. The AUC was 0.797 (p<0.001), with a cut-off value of 1.5 indicating postoperative AF with 90.9% sensitivity and 34.8% specificity (Fig.1). The overall accuracy was 62.85%, exceeding the acceptable threshold. (Table-II).

**Table-II:** Diagnostic Ratios.

Criteria	Value	95% Confidence Interval
Sensitivity	%90,90	%88,94 - %92,61
Specificity	%34,80	%31,85 - %37,84
Positive Likelihood Ratio	1,39	1,33 - 1,46
Negative Likelihood Ratio	0,26	0,21 - 0,32
Positive Prediction Value	%58,23	%57,03 - %59,43
Negative Prediction Value	%79,27	%75,54 - %82,56
Accuracy	%62,85	%60,69 - %64,97

Cut-off point: 1.50; AUC*: 0.797 (p=0.001), 95% CI*: 0.652-0.943; *AUC: Area under the curve, *CI: Confidence interval

A logistic regression model was used to evaluate predictors of postoperative AF. Among the variables analyzed, only the CHA_2_DS_2_-VASc score was a significant predictor (OR=4.33; 95% CI: 1.07-17.40; p=0.039). Comorbidities such as HT, diabetes, vascular disease, and obesity, as well as surgical technique and disease stage, showed no significant association with AF development (Table-III).

**Table-III:** Prognostic factors affecting postoperative atrial fibrillation (PAF).

Variable	β	p	OR	95% GA Lower	95% GA Top
Hypertension	1,668	0,219	5,302	0,372	75,667
DM	-0,340	0,768	0,712	0,074	6,828
Vascular disease	0,357	0,774	1,429	0,125	16,382
Obesity	-2,428	0,111	0,088	0,004	1,746
Method of Operation		0,745			
Right lower lobectomy	-18,603	0,998	0,000	0,000	---
Left upper lobectomy	-1,526	0,214	0,217	0,020	2,417
Left lower lobectomy	-1,473	0,231	0,229	0,021	2,552
Pneumonectomy	-20,617	0,998	0,000	0,000	
Stage		0,218			
Stage 1	-0,844	0,562	0,430	0,025	7,459
Stage 2	-2,145	0,075	0,117	0,011	1,244
Stage 3	2,193	0,064	8,928	7,092	90,909
CHA₂DS₂-VASc	1,466	0,039*	4,333	1,079	17,404
Constant	-1,097	0,665	0,334		

* Significant at the 0.05 level according to binary logistic regression analysis.

## DISCUSSION

In our study, the incidence of PAF following elective anatomical lung resection was found to be 11%. This is consistent with the incidence range of 10% to 20% reported in the literature and is lower than that reported in some series.^4^,^5^ The most noteworthy finding was that the CHA_2_DS_2_-VASc score emerged as a significant predictor for PAF development. This suggests that the scoring system may have prognostic value not only in cardiac surgery patients but also in patients undergoing thoracic surgery.^13^,^15^-^19^

In a previous study that included different surgical procedures, such as mediastinal mass excision and lymph node dissection in addition to lobectomy and pneumonectomy, the CHA_2_DS_2_-VASc score was used to predict the development of PAF.^4^ Unlike this study, the inclusion of only patients who underwent anatomical lung resection enabled our study to produce more specific results.

In a study by Rozencwajg and colleagues, the CHA_2_DS_2_-VASc score was shown to be associated with the total drainage volume and length of hospital stay.^20^ However, in our study, no significant relationship was found between PAF development and length of hospital stay or total drainage volume.

In the literature, parameters such as advanced age, male gender and CHF have been reported to be effective risk factors for PAF development.^6^-^8^ However, in our study, no significant effect of these variables on PAF development was detected. On the other hand, previous studies have noted that the risk of PAF development increases as the extent of resection increases (e.g., toward pneumonectomy) and similarly, in our study, the PAF rate was found to be higher in more extensive resections.^21^,^22^

Furthermore, while systematic mediastinal lymph node dissection (MLND) was performed in all oncological resections, our study design did not allow for a detailed analysis of the impact of the extent or complexity (e.g., dissection of conglomerated nodes) of MLND on PAF risk. This is a relevant factor, as aggressive hilar and mediastinal dissection can provoke local inflammation and potentially trigger arrhythmias^6^, and it should be considered in future, larger-scale studies.

Beyond predicting PAF, it is important to consider other factors that influence postoperative morbidity. Extended dissections, and inadequate postoperative pain control can contribute to a heightened stress response, sympathetic activation, and increased risk of complications, including PAF.^23^ A comprehensive pre-operative strategy addressing these factors is crucial for improving patient outcomes.

In our study, the incidence of PAF was lower in benign diseases and stage 1-2 malignant diseases compared to stage three diseases. There are insufficient data in the current literature to evaluate the effect of malignancy or disease stage on PAF development; in this context, our study contributes to the literature. Although some publications suggest that right-sided lobectomies and pneumonectomies are associated with a higher incidence of PAF than left-sided procedures, our study did not observe a significant effect of the side of the procedure on PAF development.^6^ There are studies showing that the development of intraoperative complications increases the incidence of PAF.^9^ However, since no intraoperative complications developed in the cases included in our study, our study cannot provide reliable results in this regard.

### Strength & Limitation:

The strength of this study lies in its focus on cases of PAF developing after anatomical lung resection and its evaluation of the incidence of PAF according to cancer stage. In this sense, by concentrating on a more uniform patient population, it offers a distinctive contribution to the literature. However, our findings must be interpreted in the context of several limitations. First, the retrospective and single-center design inherently carries risks of selection bias and unmeasured confounding. Second, the relatively small sample size, particularly the low number of PAF events (n=11), limits the statistical power of the multivariate analysis and the generalizability of the results. Part of the data collection period coincided with the COVID-19 pandemic, which may have affected both surgical volumes and patient follow-up. Third, as mentioned, we could not quantitatively analyze the impact of the surgical approach (VATS vs. thoracotomy) or the complexity of mediastinal lymph node dissection due to sample size constraints and the nature of the data collection. Future prospective, multi-center studies with larger cohorts are needed to validate our findings and to better elucidate the roles of surgical technique, dissection extent, and other perioperative factors in the development of PAF.

## CONCLUSION

This study demonstrates that the CHA_2_DS_2_-VASc score can be used to predict the development of PAF after thoracic surgery. This scoring system allows high-risk patients to be identified prior to surgery, enabling the planning of appropriate prophylactic approaches and contributing to the reduction of postoperative morbidity and mortality.

### Authors’ Contributions:

**HEC:** Conceived, designed and did statistical analysis & editing of manuscript, is responsible for integrity of research.

**SEA, SY and BAU:** Did data collection and manuscript writing.

**HEC, SEA:** Did review and final approval of manuscript.
